# Uniform Scale of Salaries in Poor-Law Infirmaries

**Published:** 1920-12-04

**Authors:** 


### Uniform Scale of Salaries in Poor-Law Infirmaries.

Southwark Board of Guardians are urging the Ministry of Health to take steps to establish a uniform scale of remuneration for the nursing staffs engaged in Poor-law infirmaries in order to avoid the competition which is at the present time taking place between the different authorities of these infirmaries to secure the services of the limited number of trained nurses available. This was due to an application for increased salaries from the assistant matrons, the night superintendent and the head nurses in the Southwark Hospital. The Medical Superintendent (Dr. Bruce) pointed out that they received less than they would if they came under the Whitley award of war bonus. At the same time he pointed out how unsatisfactory are the present conditions with regard to the salaries of the trained nurses employed in Poor-law infirmaries. There was a shortage in the supply of trained nurses, and it appeared as if the different London infirmaries were bidding against one another for their services. Nearly every week there appeared to be an advertisement of an infirmary offering still more money than the previous maximum. The general unfairness, unrest and discontent which resulted from this state of things was most prejudicial to the efficiency of the infirmaries, and he thought that some standard salary should be agreed on for what was practically identical work in almost identical institutions. We have long urged the necessity for a central bureau for recruiting candidates, and it is high time that an experiment was made in this direction. It would probably lead to a standardisation of probationers' salaries, which is much to be desired.

